# Antioxidant/Antibacterial Electrospun Nanocoatings Applied onto PLA Films

**DOI:** 10.3390/ma11101973

**Published:** 2018-10-13

**Authors:** Bogdanel Silvestru Munteanu, Liviu Sacarescu, Ana-Lavinia Vasiliu, Gabriela Elena Hitruc, Gina M Pricope, Morten Sivertsvik, Jan Thomas Rosnes, Cornelia Vasile

**Affiliations:** 1 Faculty of Physics, Alexandru Ioan Cuza University, 11 Carol I bvd, 700506 Iasi, Romania; 2 “P. Poni” Institute of Macromolecular Chemistry, Romanian Academy, 41A Grigore GhicaVoda Alley, 700487 Iasi, Romania; livius@icmpp.ro (L.S.); vasiliu.lavinia@icmpp.ro (A.-L.V.); gabihit@icmpp.ro (G.E.H.); cvasile@icmpp.ro (C.V.); 3 Veterinary and the Food Safety Laboratory, Food Safety Department, 700489 Iasi, Romania; ginacornelia@yahoo.com; 4 Nofima AS, Deptartment of Processing Technology, Muninbakken 9-13, Tromsø 9291, Norway; thomas.rosnes@nofima.no

**Keywords:** electrospinning, nanocoating, chitosan, vegetable oil, essential oil, cold-press oil, antimicrobial, antioxidant

## Abstract

Polylactic acid (PLA) films were coated by coaxial electrospinning with essential and vegetable oils (clove and argan oils) and encapsulated into chitosan, in order to combine the biodegradability and mechanical properties of PLA substrates with the antimicrobial and antioxidant properties of the chitosan–oil nanocoatings. It has been established that the morphology of the electrospun nanocoatings mainly depend on the average molecular weight (MW) of chitosan. Oil beads, encapsulated into the main chitosan nanofibers, were obtained using high-MW chitosan (Chit-H). Oil encapsulated in chitosan naoparticles resulted when low-MW chitosan (Chit-L) was used. The coating layer, with a thickness of 100 ± 20 nm, had greater roughness for the samples containing Chit-H compared with the samples containing Chit-L. The coated PLA films had higher antibacterial activity when the nanocoating contained clove oil rather than when argan oil was used, for both types of chitosan. Nanocoatings containing Chit-H had higher antibacterial activity compared with those containing Chit-L, for both types of oil tested, due to the larger surface area of the rougher nanoscaled morphology of the coating layer that contained Chit-L. The chitosan–clove oil combination had higher antioxidant activity compared to the simple chitosan nanocoating, which confirmed their synergistic activities. The low activity of systems containing argan oil was explained by big differences between their chemical composition and viscosity.

## 1. Introduction

Due to increased demand for food packaging, efforts are being made to increase the storage- and shelf-life of food products by developing active antimicrobial packaging that does not require the use of synthetic additives [1]. The antibacterial agent may be incorporated into the packaging material [2] or coated onto it [3,4].

Various natural antibacterial agents have been encapsulated into different matrices to create coatings onto base packaging materials, such as cinnamaldehyde and carvacrol encapsulated in soy protein isolates [5]; nisin and natamycin in polyvinylchloride lacquer [6]; sorbic acid in polyvinyl acetate [7]; essential and vegetable oils, such as garlic oil and rosemary oleoresin, in soy protein isolate [8]; and oregano essential oil in ethylene-vinyl alcohol copolymer film [9].

Due to its well-known antibacterial activity against various pathogens such as *Klebsiella pneumoniae*, *Escherichia coli*, *Staphylococcus aureus*, and *Pseudomonas aeruginosa* [10], chitosan has been extensively studied to obtain antibacterial coatings [11]. Chitosan was also used to encapsulate another antibacterial agent, nisin [12], which, in combination with chitosan, could improve the antimicrobial activity of the coatings [13]. The biodegradable chitosan is non-toxic and confers antibiofilm, antioxidant, and antifungal properties to the coated layer [14].

Various procedures have been proposed for preparation of the antimicrobial coating: Spreading with a thin-layer chromatography applicator [5] or a lab bench coater [7]; using a bar coater [15] or a brush [16]; spraying (nebulization) [17]; lamination [18]; gravure printing [9]; dip-coating [19]; and translated, at industrial scale, by coupling with a wet-coating station and roll-to-roll system [20].

In this work, electrospinning/electrospraying is used as the coating technique. The properties of the coatings, containing the essential and cold-pressed oils, depend on the way they are distributed onto the base polymer (substrate) and also on the way they make contact with the food. In this respect, the nanoscale dimensions and high area-to-volume ratio of the electrospun/electrosprayed nanostructures (nanofibers and nanoparticles), help to improve the contact between the coatings and the packed food, while the encapsulation of the essential and vegetable oils, into the polymeric nanostructures, helps to maintain their antibacterial and antioxidant activities over a longer time period. Therefore, electrospinning/electrospraying is an effective and convenient method to obtain nanocoatings [21] due to several advantages offered by this technique, such as: 

(a) By electrospinning, very thin nanofibers with a high porosity and area-to-volume ratio can be obtained.

(b) The thickness of the coated (deposited) layer can be easily controlled by changing the deposition time or the flow rate of the electrospinning solutions. Thus, it is possible to obtain a very thin coating (nanocoating) with a very small quantity of materials, which in many cases is enough to obtain the desired antibacterial and antioxidant [4] or antifungal activity [22]. In comparison, with the coating thickness obtained by other methods, such as solvent casting or dip-coating with special film applicators (3 μm [23], 2 ÷ 9 μm [6], 2 ÷ 3 μm [24]), the very thin layer (100 nm or even less) has the added advantage of requiring a low amount of the coating material.

(c) Plasticizers are often added to the chitosan coating layer [12] to overcome the brittleness exhibited during the package deformation, and to improve the flexibility and processability. As a coating layer, the nanofibers can exhibit better ductility than the corresponding bulk material [25] due to low-nanofiber crystallinity, which resulted from rapid solidification of the ultrafine electrospun jets [26]. Thus, it is expected that the nanosized electrospun coating layers will exhibit the needed flexibility, either in the form of nanofibers or nanoparticles, without the plasticizer addition.

In the present work, the polylactic acid (PLA) films were coated by electrospinning with bio-formulations containing chitosan and one of the two vegetable oils (i.e., clove and argan oils), which were chosen due to their different compositions. According to the literature data, acylglycerols constitute 99% of the argan oil composition, while the unsaponifiable matter contains tocopherols, squalene, sterols, and phenols as the main antioxidant compounds [27,28]. Eugenol (~80%) is the main volatile component of clove oil, which is responsible for its strong antioxidant and antimicrobial activities [29]. The different compositions of the two types of oils are expected to result in different antimicrobial and antioxidant activities of the coated PLA films, and also in different rheological properties, which, in turn, will determine differences in the morphologies of the electrospun coated layers at nanoscale level.

PLA films were coated with active bio-formulations with the intention to combine the biodegradability and mechanical properties of PLA substrate polymer with the antimicrobial and antioxidant activities and biological functions of clove and argan oils, in combination with the chitosan. The oils were encapsulated in chitosan by coaxial electrospinning so as to prolong their functions as antimicrobial and antioxidant agents over a long time, to prevent their degradation and loss during the processing conditions, and also to prevent their rapid diffusion and migration into the packaged food.

## 2. Materials and Methods 

### 2.1. Materials

Chitosan with two different average molecular weights (MW) was purchased from Sigma-Aldrich (Schnelldorf, Germany): Highly viscous chitosan (Chit-H), MW = 310,000–375,000 g/mol, deacetylation degree DD ≈ 77% [21], with a dynamic viscosity in 1% acetic acid (20 °C) > 400 mPa·s; and low viscous chitosan (Chit-L), MW = 50,000–190,000, DD ≈ 87% [21], with viscosity in 1% acetic acid of 20–300 cP. The range values for MW (average molecular weight) were taken from producer specifications. 

Glacial acetic acid (analytical purity) and chloroform were obtained from Chemical Company (Iasi, Romania).

Vegetable oils (argan and clove oils) with antioxidant and antimicrobial activities were chosen and were purchased from the Fares SA (Orastie, Romania) and Herbavit SA (Oradea, Romania) companies, respectively. The phenol content of the oils were 1.16 mg GAE/g DW (gallic acid equivalents/dry weight) for the clove oil and 0.02 mg GAE/g DW for the argan oil.

Two types of PLA films were coated: (a) Hot-pressed films obtained from PLA 2002D pellets (from NatureWorks LLC, Minnetonka, MN, USA), and (b) commercial NATIVIA^®^ NTSS 40 µm PLA foils (from Taghleef Industries, Newark, DE, USA). The hot-pressed PLA substrates with a thickness of 0.30 ± 0.05 mm were obtained using a Carver press at 175 °C (2 min pre-melting and 2 min pressing at 240 bar). The NATIVIA^®^ commercial PLA foils (Taghleef Industries, Newark, DE, USA) were used in order to test the applicability of this coating method for commercial products that currently exist in the market.

### 2.2. Preparation of the Nanostructured Coatings

**Figure 1:**
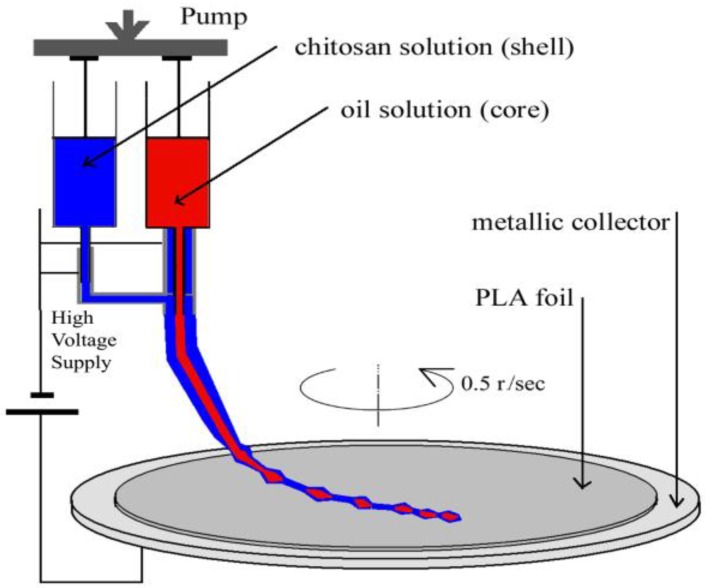
The coaxial electrospinning set-up used for coating the polylactic acid (PLA) foils. The PLA foil is placed directly onto the metallic collector.

The electrospinning system, used for coating the PLA foils (Figure 1), consisted of a high voltage supply (HV), a rotating metallic-plate collector, and two syringes with a coaxial needle oriented perpendicular to the metal plate. The PLA substrate film was placed on the metallic collector. The high direct voltage (0 to 30 kV) was applied between the metal plate and the metallic needle.

The low surface energy of polymeric substrates often leads to poor adhesion of the coatings to the substrates. To improve the adhesion, the surface is usually activated or functionalized by corona [9] or plasma [30] treatment. Before getting coated with the chitosan–oil formulations by electrospinning, the PLA substrates were treated by exposure to cold plasma (nitrogen gas discharge atmosphere, 1.3 MHz frequency, 100 W power, 0.4 mbar pressure). Plasma treatment, and the consequent air exposure, leads to the implementation of some functionalities on the surface, such as groups and free radicals containing nitrogen and oxygen, which are able to interact with functional groups of the components of the nanocoating that were deposited by electrospinning [31].

After plasma treatment, the coaxial electrospinning was applied to obtain oil-loaded nanostructures, by encapsulating the clove or argan oil into the chitosan nanostructures, simultaneous with being coated onto the plasma-treated PLA substrates. The coaxial system consisted of chitosan supplied to the outer nozzle and oil solutions supplied to the inner nozzle. 

For the hot-pressed PLA film substrates, the samples were designated the codes HC, HA, LC, and LA where H and L signifies high- and low-viscous chitosan, respectively, and C and A signifies clove and argan oil, respectively.

Correspondingly, for the commercial NATIVIA^®^ NTSS substrate, the samples were labelled as HC-NATIVIA and HA-NATIVIA. Additionally, H-NATIVIA was designated the commercial NATIVIA^®^ NTSS 40 µm, coated only with Chit-H, and PLA-NATIVIA was designated the uncoated PLA commercial foil (Table 1).

Both of the chitosan and oil solutions had the same concentration of 1.5 wt%. Chitosan was dissolved in 9:1 glacial acetic acid and water, respectively, argan oil was dissolved in chloroform, and clove oil was dissolved in glacial acetic acid. Electrospinning parameters (for all samples) were 1 kV/cm electrical field strength, 1.2 μL/min feed rate (for both the inner and outer needle), and 30 min deposition time. To be sure of complete solvent removal, the electrospun nanostructures were placed for two days in a vacuum desiccator (Binder GmbH, Tuttlingen, Germany). The absence of the acetic acid in the deposited mesh in was confirmed by IR spectroscopy (using a Bruker VERTEX 70 spectrometer, Ettlingen, Germany).

### 2.3. Investigation Methods

Scanning electron microscopy (SEM/EDX) analyses were carried out using a QUANTA 200 scanning electronic microscope (Thermo Fisher Scientific, Waltham, MA, USA) with an integrated (EDX system, (Thermo Fisher Scientific Waltham, Waltham, MA, USA), and a GENESIS XM 2i EDAX with SUTW detector (FEI Company, Eindhoven, The Netherlands).

Atomic force microscopy (AFM) investigations were done with a Solver-Pro-M type instrument (Solver Inc., Moscow, Russia) under ambient conditions, using standard tips of Si_3_N_4_ (10 nm curvature radius). The root mean square roughness was calculated from the total image sample after a second-order flatness treatment of the raw data. NT-MDT Nova v.1.26.0.1443 software (NT-MDT Spectrum Instruments, Moscow, Russia) was used for the acquisition and analysis of the images.

Transmission electron microscope (TEM) investigations were conducted with a Hitachi High-Tech HT7700 microscope (Hitachi High-Tech GLOBAL, Tokyo, Japan) high contrast mode at 100 kV accelerating voltage) on electrospun grids (300 mesh holey carbon coated copper grids). The grids were placed onto the PLA substrate during the electrospinning process.

Antimicrobial activity was determined, using two kinds of tests, in order to verify the differences between the antimicrobial activities of the clove and argan oils.

In the first test the suspension and culture medium were prepared as is described in the ISO 22196 (ISO 22196: Plastics—measurement of antibacterial activity on plastics and other non-porous surfaces (2011)). The test strains were incubated for 24 h at 37 °C. The antimicrobial testing method consisted of placing a drop of a suspension, of either *Escherichia coli* (ATCC 8739) or *Staphylococcus aureus* (ATCC 6538), onto the surface of the tested material (with a surface area of 5 cm × 5 cm). Each tested sample was prepared in a separate sterile vessel, with the test surface facing upwards. Then 0.4 mL of the test inoculum was dropped onto the tested surface. The test inoculum was covered with a piece of film (with a surface area 4 cm × 4 cm), which did not have anti-bacterial properties (neutral film), and was pressed mildly onto the film so that the test inoculum spread to the periphery. Half of the untreated specimens were processed instantly after inoculation, by adding 10 mL of Soybean Casein Lecithin Polysorbate Medium (SCDLP) broth (ISO 22196) to the vessel containing the test specimen. After this, the sum of viable bacterial cells was assessed. This obtained amount was used to evaluate the recovery rate of the bacteria from the investigated test specimens. After the inoculation of the specimen and the application of the cover film, the lid of the vessel was changed. After a 24 h incubation period, at 35 °C, the bacterial suspension was released from the coverslip-test sample and the sum of the viable bacterial cells that had survived was determined. The log values were the mean of 3 parallel samples (for each film type). The log reduction was the difference between the growth obtained with a neutral film (Stomacher bag) and the growth obtained with an active treated film. Sometimes a reduction, rather than growth, was observed with the neutral film, because of the low pH of the free lactic acid containing the PLA.

The second test was performed as it is described in the ISO 16649-2/2007: Microbiology of alimentary and animal products. There are several phases in the procedure for testing antimicrobial activity against *Escherichia coli*, *Salmonella typhymurium*, and *Listeria monocytogenes*: Sterilization of samples; inoculation with ATCC culture bacteria; 24 and 48 h (at 44 °C) inoculation and incubation, respectively; and the identification of target germs. Sterilization of the samples was performed for 20 min at 0.5 bar and at 110 °C, in an autoclave. Formation of the ATCC cultures was performed by seeding the average pre-enrichment, followed by incubation for 24 h at 37 °C; counting the number of colonies in the 0.1 mL culture after the culture medium separation; and then seeding 0.1 mL of bacterial culture ATCC, using sterile swab samples taken from the surface. The following standardized bacteriological procedures were employed in order to identify the target germs: For *Escherichia coli*, the SR ISO 16649, horizontal method for beta-glucuronidase-positive *Escherichia coli* quantification, was used. Then colonies were counted at 44 °C using 5-bromo-4-chloro-3-indolyl beta-d-glucuronide according to Minerals Modified Glutamate Broth (Cat. 1365), which produce blue or green–blue colonies on agar glucuronide; for *Listeria monocytogenes,* SR EN ISO 11290 was used; and for *Salmonella* sp., SR EN ISO 6579/2003/AC/2004/AC/2006, Amd.1:2007 was used.

The antioxidant activity of PLA (NATIVIA) coated films was measured by DPPH (1,1-diphenyl-2-picryl-hydrazyl) free radical assay [32]. The samples (coated films) were immersed in the DPPH solution and the decrease of the absorbance at 517 nm, which reflected the amount of DPPH radicals in the solution, was measured by means of a Cary 60-UV-Vis Spectrophotometer (Mettler Toledo, Columbus, OH, USA). The measurements were done at different immersion times (after 0, 15, 40 and 60 h immersion time). The DPPH solution (in the absence of the coated films) was used as a control. The radical scavenging activity (RSA) of the samples was expressed by the relative decrease in DPPH absorbance at 517 nm (RSA = 1−A_SAMPLE_(t)/A_DPPH_(t) where A_DPPH_(t) and A_SAMPLE_(t) are the absorbance of the control and sample at the time of measurement). For testing, a 4 cm^2^ area of the coated film was cut into pieces and immersed in 1.5 mL of DPPH solution in methanol with a concentration of 8 micrograms/mL. Before immersion and testing, the samples were stored for seven days at room temperature. Between measurements the solutions were stored in the dark at 24 °C.

## 3. Results and Discussion

### 3.1. Transmission Electron Microscope (TEM) Results

**Figure 2:**
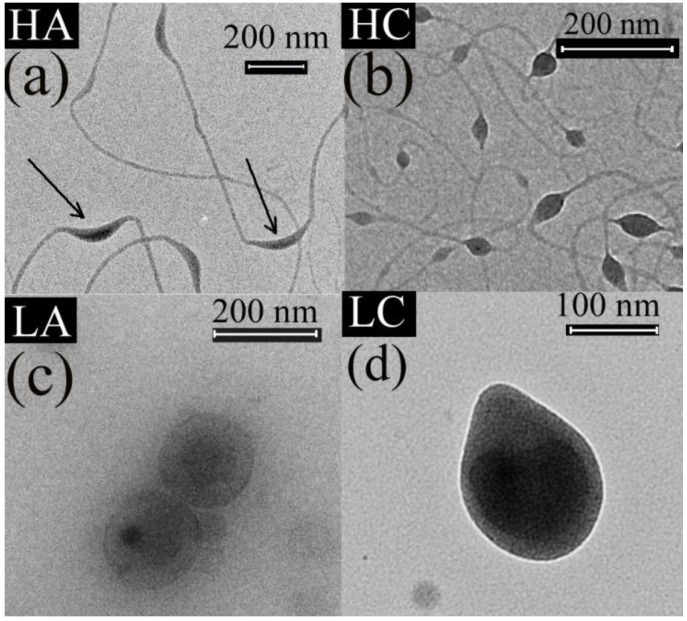
Transmission electron microscope (TEM) images of the studied samples: (**a**) HA; (**b**) HC; (**c**) LA; and (**d**) LC samples.

The TEM images show a hybrid fiber and particle morphology for the HA and HC samples (Figure 2a,b), and particle morphology for the LA and LC samples (Figure 2c,d).

It is well known that sufficient chain entanglements should occur in the electrospun solution [33] in order to obtain nanofibers by electrospinning. Thus, the presence of fibers for the HA and HC samples could be explained by the high MW of chitosan, which resulted in better macromolecular entanglements in the electrospun solution. Due to the low MW, the chain entanglements in LA and LC samples were not enough [34] to sustain the fiber formation, which resulted in the spraying of beads (which ultimately may form a rather compact film). Thus, the only spinnable (nanofiber mesh forming) samples are those containing Chit-H.

The TEM images also show two different encapsulation types, of the vegetable oils into the chitosan, depending on the MW of the chitosan used for the shell solution: The LA and LC samples (Figure 2c,d), with low-MW chitosan, had a particle morphology whereby isolated oil beads were encapsulated by chitosan; whereas the HA and HC samples (Figure 2a,b), with high-MW chitosan, had oil encapsulated as scattered beads along the main chitosan fiber.

The beads-into-fiber structure of the HA and HC samples can be explained by the different molecular structure and viscosities of the shell and core solutions used in the coaxial system, that is, a highly viscous (>400 mPa·s) electrospinnable macromolecular (Chit-H) shell solution and a sprayable oil core solution with very low viscosity (1–2 mPa·s). It is known that the similarity between the rheological properties of the core and shell solutions control the viscous dragging exerted by the shell solution on the core solution [35]. If the viscosity of the core solution is too low, compared with the shell solution, the viscous drag exerted by the shell solution is unable to overcome the cohesive forces of the core solution [36]. This was the case for the solutions used for the HA and HC samples. The entanglements provided by the highly viscous shell Chit-H solution were enough to prevent fiber rupture, which can be caused by low viscosity of the core oil solution, but, due to the low viscosity, the core oil solution was unable to follow the stretching of the macromolecular shell solution [37], thus taking the form of beads into the chitosan fibers [38]. It can also be observed that the HA and HC samples had a similar fiber diameter (10–20 nm), which supports the assertion that the HA and HC fibers were mostly made of Chit-H and the oil was encapsulated into the beads along the fibers. Similar morphologies, with beads-into-fiber structures, were obtained by other authors for proteins [39] or dexamethasone [40] encapsulated along the polymeric nanofibers as beads, which can act as depots for sustained drug release [40]. Other authors reported that coaxial electrospining employed to encapsulate sunflower oil into PEO produced beaded fibers, with the oil encapsulated into the beads [36]. The authors also reported that the oil was confined or encapsulated solely inside these beads [36]. This is in good agreement with our findings, that is, the fibers contained mainly chitosan and the oil was distributed as beads along the main chitosan fiber.

From the TEM images, another observation can be made regarding the morphology of the HA and HC samples: The beads distributed along the Chit-H fibers were much more elongated for the HA sample (Figure 2a) compared with the HC sample (Figure 2b). Because a higher viscosity of the core fluid increases the viscous drag, exerted by the shell solution onto the low molecular core fluid [36], the more elongated argan oil beads can be explained by the higher viscosity of the argan oil compared with the clove oil. Considering the rapid evaporation of the solvent during the jet stretching [41], it is more relevant to compare the viscosities of pure oils instead of the oil solutions. In our case, argan oil was ten times more viscous (~0.6 poise [42]) than the clove oil (~0.06 poise [43]), which explains the more elongated argan oil beads.

### 3.2. Atomic Force Microscopy (AFM) Results

**Figure 3:**
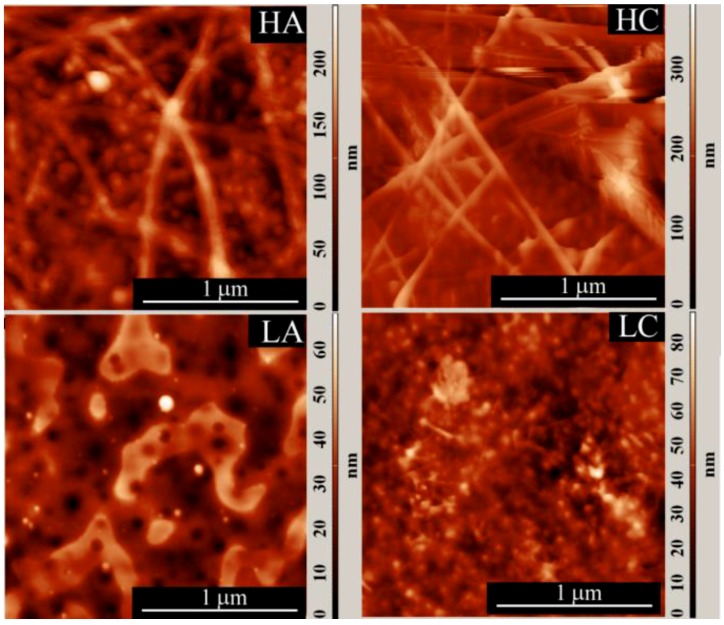
Atomic force microscopy (AFM) images of the studied samples: (**a**) HA; (**b**) HC; (**c**) LA; and (**d**) LC samples.

The particle and fiber diameters evaluated from AFM images (Figure 3) are all in the nanometer scale with similar values as those revealed by TEM images (Table 2).

**Figure 4:**
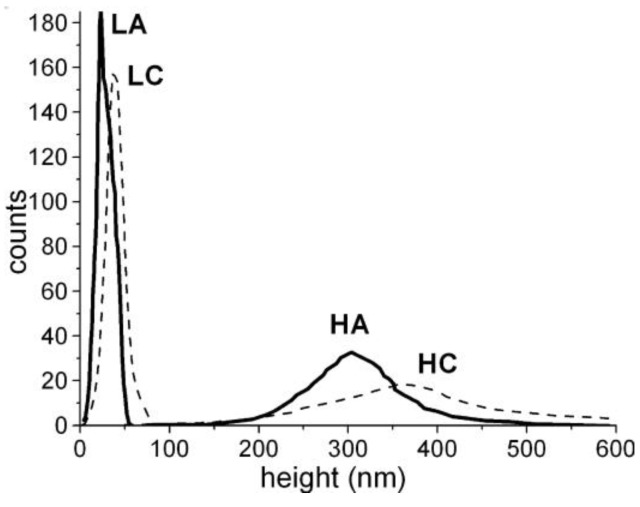
AFM height distributions for the studied samples: (**a**) HA; (**b**) HC; (**c**) LA; and (**d**) LC samples.

**Figure 5:**
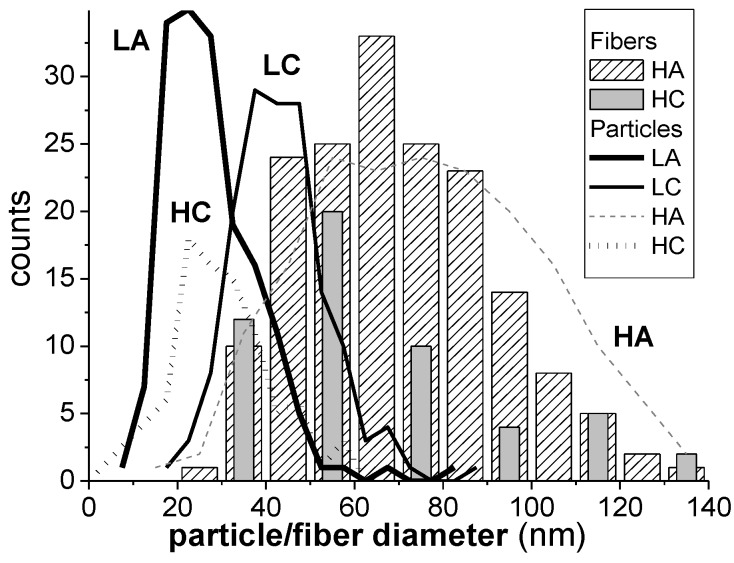
Distributions of the particle (lines) and fiber (bars) diameters, in the coated layers, determined from AFM.

It is interesting to compare the width (range) of the particle and fiber diameter distributions, determined from the AFM images, with the width of the AFM height distribution (Table 2, Figure 4 and Figure 5). The width of the AFM height distribution was similar to the width of the particle AFM diameter distributions for the LA and LC samples, but was much wider than the particle and fiber diameter distributions for the HA and HC samples.

This is because, compared with the HA and HC samples, the LA and LC samples had a more compact particle arrangement, with smaller voids between them, which narrowed the height distribution. This was also shown by other authors [44,45], who correlated the roughness (or the width of the height distribution) of nanoparticle layers with the experimentally measured nanoparticle sizes, and also with the way the particles are packed [44]. For a closely (or orderly) packed spherical structure, with spheres tangent to one another (particle-near-particle), the roughness is low and the AFM height distribution approaches the particle diameter distribution (roughness approaches the average diameter of the particles). But when the same particles are randomly packed in a more porous arrangement, the roughness increases [45]. Thus, it can be concluded that, for the LA and LC samples, the structures were more compact with lower porosity (smaller voids between particles) and, consequently, with narrower height distribution than the HA and HC samples.

### 3.3. Scanning Electron Microscopy (SEM) Results

**Figure 6:**
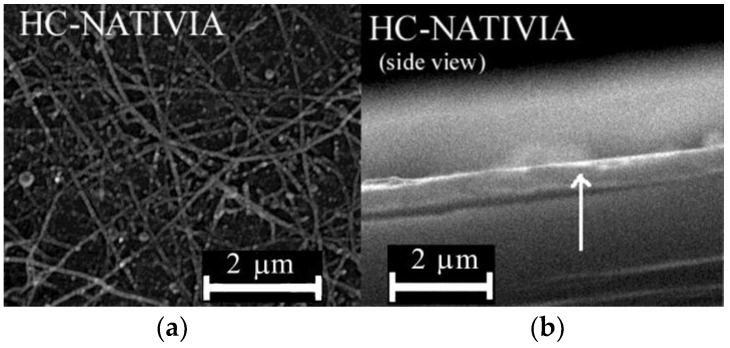
Scanning electron microscopy (SEM) images of the HC-NATIVIA sample: (**a**) Morphology of the coated surface; and (**b**) side view showing the coated layer.

The SEM images for the HC-NATIVIA coated sample show the presence of nanofibers with diameters around 60–70 nm (Figure 6a). In contrast with the NATIVIA^®^ PLA commercial foils, the hot-pressed PLA films experienced sample damage due to high electron acceleration during the SEM analysis, which lowered the resolution of the SEM images. Based on the presence of nitrogen, the SEM-EDX analysis was used to investigate the presence of chitosan on the PLA substrate. The mapping of nitrogen distribution, with an average nitrogen content of 5–6%, revealed a uniform coating on the surface at a micrometric scale.

Because of their smooth and glossy finish, the PLA-NATIVIA^®^ films were used to evidence the coated layer. The side view of the coated PLA-NATIVIA^®^ foil (Figure 6b) shows the coating layer with a thickness of 100 ± 20 nm. Considering that the entire output of the electrospinning syringe was deposited onto the PLA-NATIVIA^®^ foil (10 μg/cm^2^), based on chitosan and oil density, we would need to have the coated layer at a thickness of around 250 nm for maximum compactness of the chitosan (with no porosity of the coated layer). These results, therefore, show that only a part of the syringe output was deposited onto the PLA substrate (about 4 μg/cm^2^ assuming no porosity of the chitosan mesh). Considering porosity (void percent) of ~80% for the chitosan mesh [46], results suggest that the amount of the antimicrobial oil deposited onto the PLA substrate was at least 0.4 μg/cm^2^. For meat packaging applications, the contact between the antibacterial coated layer and the meat packaged is realized by means of the moist layer, which exists between the packaging foil and the meat. If we consider a moist layer with a thickness of approximately 1 mm is in contact with the meat, the oil concentration throughout the moist layer would be ~4 μg/mL, which is well above the minimum inhibitory concentration of clove oil against *E. coli* (0.1 mg/mL) [47].

Therefore, nanostructured chitosan morphologies were obtained by electrospinning, with clove and argan oils encapsulated as beads into the chitosan nanofibers or nanoparticles. The nanofibres were predominant in the samples obtained from the electro-spinnable Chit-H. Nanoparticles were obtained when Chit-L was used. 

### 3.4. Antibacterial Tests

The results of the antibacterial tests are presented in Table 3 (ISO 22196) and the Table 4 (ISO 16649-2/2007). 

The results of the two antibacterial tests are presented as log reduction of the viable cells (first test—Table 3) and as inhibition percent (second test—Table 4). Regarding the efficiency of the coating procedure, it can be noted that there was higher antibacterial activity in the coated versus uncoated samples (Table 3), and also higher antibacterial activity on the chitosan–oil coatings versus chitosan coatings (Table 3 and Table 4). Regarding the two types of chitosan and oils used for the coatings, the second antibacterial tests also showed higher antibacterial effect for Chit-H versus Chit-L, and for clove oil versus argan oil (Table 4).

More specifically, for both Chit-H–argan oil and Chit-H–clove oil combinations, the results obtained by the first test (Table 3) evidenced improved antibacterial activity of the coated PLA films compared with the uncoated films (for both NATIVIA and hot-pressed PLA films)*.* Even the HA-NATIVIA sample (with 0 log reduction) had higher antibacterial activity against *S. aureus* (Table 3) compared with the uncoated NATIVIA film (with negative log reduction, i.e., cell proliferation). In the case of *E. coli* bacteria, antibacterial effect was observed even for the uncoated NATIVIA films because of the decreased pH by the free lactic acid, which was present in the film (Table 3).

Also, both tests showed that the combination Chit-H–oil had higher antibacterial effect than Chit-H coated alone (Table 3 and 4) and, therefore, indicates that improved antibacterial activity could be obtained by coating the PLA films with chitosan. In addition, the coaxial encapsulation of the clove and argan oils, electrospun together with the chitosan, led to enhanced antimicrobial activity for both NATIVIA and hot-pressed PLA films.

The second antibacterial test also showed higher antibacterial effect for Chit-H versus Chit-L, and for clove oil versus argan oil (Table 4).

The different antibacterial activities of the two types of oils can be explained by the different amounts of the compounds responsible for the antimicrobial activity in the two oils (primarily phenolic compounds [48,49,50]). Eugenol, which increases antimicrobial and antifungal activity, is the main component (~80%) of clove oil. On the contrary, the phenolic compounds in argan oil are found in much lower amount (<1%), which explains the lower antibacterial and antifungal activity of argan oil compared with clove oil [51,52].

The higher antibacterial activity of Chit-H compared with Chit-L cannot be explained by its higher MW or by its lower deacetylation degree (DD) because many authors reported an opposite result (i.e., lower antibacterial activity for higher MW and lower DD), as it is shown further on. 

Indeed, it is reported in literature that chitosan had lower antibacterial activity for higher MWs [53,54], for both gram-positive bacteria (GP-b) [55] and gram-negative bacteria (GN-b) [56]. This effect is reported to take place for MWs higher than 100 kDa [57], and some authors even report low activity for MWs > 30 kDa [58]. As the MW of the chitosan types used in this work was higher than this threshold (50–200 kDa for Chit-L and 300–370 kDa for Chit-H) [57,58], we conclude that the higher antibacterial activity of Chit-H (for the same type of oil) cannot be attributed to its higher MW. 

Moreover, the different DD for the two types of chitosan cannot explain the higher antibacterial activity for the Chit-H (for the same type of oil). There are many papers reporting higher antibacterial activity for higher DD of chitosan [59,60,61,62,63,64], due to the disruption of the cell wall which is caused by the higher number of free amino groups present in chitosan with higher DD [65]. Our results, showing lower antibacterial activity for the chitosan with higher DD (DD ≈ 87% for Chit-L compared to DD ≈ 77% for Chit-H) are opposed to the reports presented above, and also to another report which showed higher antibacterial activity for chitosan with higher DD and lower MW [66].

The higher antibacterial activity of Chit-H compared with Chit-L can be explained by the larger surface area of the rougher nanoscaled morphology of the coating layer for Chit-H samples compared with the Chit-L samples (according to SEM, TEM and AFM results).

In other papers it was reported that the chitosan nanoparticles (MW of 680 kDa) exhibit higher antibacterial activity against *E. coli* than the corresponding microparticles obtained from the same chitosan [56], because of the higher surface charge [67] and the larger surface area of the nanosized chitosan nanoparticles, which probably tightly adsorbed onto the bacteria cells surface, disrupting the normal functions of the membrane [56].

There are also studies showing that the bacterial attachment decreases when the exposed substrate surfaces (such as polydopamine [68]) have higher roughness [69,70], for both gram-positive bacteria (GP-b) and gram-negative bacteria (GN-b) [71]. Some authors have found that GP-b (which has a more rigid membrane than GN-b) are more sensitive to roughness than GN-b, due to the surface nano-irregularities, which limit the number of anchoring points for GP-b bacteria. In this way, the surface area in contact with the membrane is reduced, which in turn reduces the adhesion of GP-b to the rougher surface [72,73]. Additionally, it was hypothesized that air entrapped by the topographical features inhibited contact between *S. aureus* and the substrate [74]. Our results agree with these reports. Furthermore, among Chit-H samples, the GP-b *L. monocytogenes* (for the samples HA, HA-NATIVIA, and HC-NATIVIA after 48 h) had maximum inhibition. This confirms the above-mentioned results, which state that the GP-b are more sensitive to roughness, and make us conclude that the higher antibacterial activity of the Chit-H samples was attributed to the higher roughness of the coating.

### 3.5. Antioxidant Activity

It is known that it is advisable to perform the antioxidant tests with fresh DPPH solutions. However, if prolonged experiments are required (as are further presented in this section), the DPPH test can also be performed with solutions prepared tens of hours before testing without a substantial reduction in the activity of DPPH [75]. In order to test the stability of our DPPH solution, the absorbance of the DPPH (control) solution was measured over time. After 70 h (solution stored in the dark) the absorbance of the DPPH solution was about 80% of the initial value (with an almost linear decrease). This result is in line with other experimental results of other authors, which show that, after 24 h post preparation, the free radical activity of 0.1 mM DPPH methanolic solution was still very similar to that of the fresh preparation (about 20% activity was lost after about 120 h) [75].

It has been previously shown that it is possible to evaluate the antioxidant capacity of various compounds without preliminary solvent extraction. The reaction between the radical and the antioxidant occurs at the interface, when they come into contact, or by liquid diffusing into the interior of the reacting solid [76,77]. Using this method, it was possible to measure the antioxidant activity of solid matrices containing polyphenols [78], which are known to be found in argan [79] and clove oils [80]. In our study a similar approach was used: A small area of the coated films was cut into pieces and immersed into the methanolic DPPH radical solution. The antioxidant activity of the coated films was expressed by the relative decrease in DPPH absorbance at 517 nm:

Relative Scavenging Activity (RSA) = 1 − A_SAMPLE_(t)/A_DPPH_(t) (1)

In order to eliminate the effect of decreasing DPPH absorption over time, the control was considered the absorbance of the control DPPH solution at the time of spectrophotometric analysis (A_DPPH_(t)).

In the first test, a 1 cm^2^ area, from the HC-NATIVIA and HA-NATIVIA freshly coated films, was cut into pieces and immersed for 24 h in 1.5 mL of DPPH solution in methanol (8 μg/mL). This first test evidenced the higher antioxidant activity of the samples containing clove oil. The RSA values were 0.29 for the HC-NATIVIA sample and 0.17 for the HA-NATIVIA sample. These results were in good agreement with the higher phenolic content in clove essential oil compared with argan oil. Due to high eugenol content, the concentration required to scavenge 50% of 2,2’-azino-bis 3-ethylbenzthiazoline-6-sulfonic acid (ABTS) free radicals (half inhibitory concentration) was much higher for argan oil (~5800 µg/mL) compared with clove oil (~8 µg/mL) [81]. At the same time, taking into consideration the low volatility and diffusivity of the components of argan oil (fatty acids), a very low antioxidant and antimicrobial activity of argan oil was expected.

**Figure 7:**
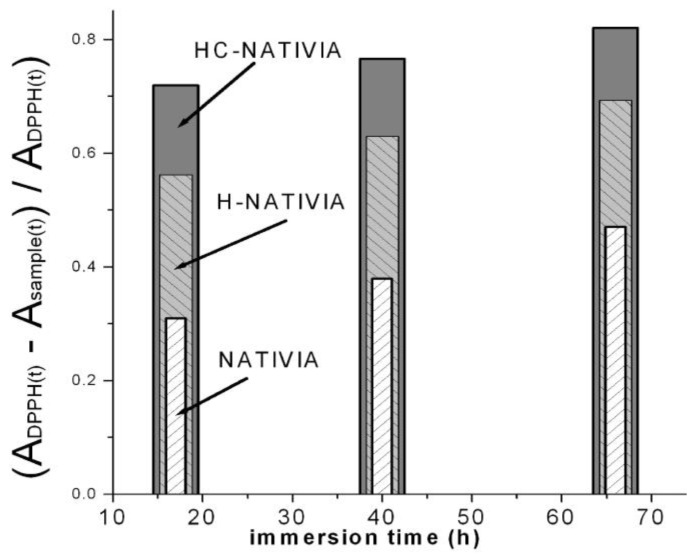
Antioxidant activity versus immersion time for the NATIVIA^®^ films coated with Chit-H–clove oil (HC-NATIVIA), Chit-H (H-NATIVIA), and the uncoated film (NATIVIA).

In a second test, the sample with higher antioxidant activity (HC-NATIVIA) was chosen to measure the antioxidant activity over the immersion time. Before testing, the samples were stored for seven days at room temperature, in order to check the encapsulation and persistency of the volatile clove oil in the samples. The results were compared with the corresponding uncoated (NATIVIA) and chitosan coated film (H-NATIVIA). In this second test, a higher film area (4 cm^2^) was cut and immersed into the same amount of radical solution with the same concentration. It can be observed that all the three samples had antioxidant activity (Figure 7) that was maintained over a fairly long time (tens of immersion hours) as a result of the clove oil encapsulation into the chitosan fibers. The concentration of biphenyl radicals continued to decrease (RSA increased) in the solution containing the immersed film relative to the control sample, even after 70 h of immersion. The antioxidant activity of the uncoated NATIVIA, which was lowest among the tested samples, was due to the residual lactic acid in the films.

Of these three samples, PLA-NATIVIA^®^ films (HC-NATIVIA) coated with chitosan–clove oil had the highest antioxidant activity. In spite of the high volatility of the clove oil [81,82], the antioxidant activity of the HC-NATIVIA was maintained even after seven days of shelf storage. This shows that clove oil can be efficiently immobilized into the coating layer by encapsulation in chitosan. Therefore, the antioxidant tests confirmed the TEM results and the presence of the encapsulated clove oil into the chitosan fibers, which results in a combined effect obtained by simultaneous use of chitosan and clove oil.

## 4. Conclusions

The novelty of this study lies in providing a new and effective approach of nanocoating polymeric films with chitosan by coaxial electrospinning, with the advantage of simultaneous encapsulation of active vegetable and/or essential oils into the chitosan.

Polylactic acid (PLA) films were coated by coaxial electrospinning with formulations containing essential or vegetable oils (clove and argan oils, respectively) and encapsulated into chitosan in order to obtain biodegradable packaging materials with antimicrobial and antioxidant properties. When chitosan with high MW (Chit-H) was used, the coaxial electrospinning produced beaded chitosan nanofibers, with the oil distributed and encapsulated along the main chitosan fiber. Chitosan nanoparticles encapsulating the oil were obtained with low-MW chitosan (Chit-L). The roughness of the coating layer was higher for the samples prepared with Chit-H compared with samples prepared with Chit-L. The PLA films coated with chitosan–oil formulations had higher antibacterial activity than the films coated only with chitosan. The clove oil had higher antibacterial activity than the argan oil, for both types of chitosan, because of its higher phenolic content. Chit-H samples had higher antibacterial activity compared with Chit-L, for both types of oil, due to the higher specific surface area of the rougher nanofibrous morphology of the coating layer. As was expected, due to the phenolic content of clove oil, the chitosan–clove oil combination had higher antioxidant activity compared to the simple chitosan coating. Considering the volatility of the clove oil, its immobilization into the coating layer can be realized by encapsulation in chitosan. The TEM results and the antioxidant tests also confirm the encapsulation of the oil into the chitosan.

## Figures and Tables

**Table 1:** PLA samples uncoated and coated with chitosan and clove oil or argan oil, using the electrospinning technique.

Code	Sample Description
	**Uncoated Samples**
PLA Hot-Pressed	Hot-pressed PLA films obtained from PLA 2002D pellets (NatureWorks LLC)
PLA-NATIVIA	Commercial NATIVIA^®^ NTSS 40 µm PLA foils (from Taghleef Industries)
	**Coated Samples: PLA Hot-Pressed Substrate**
PLA-H	PLA hot-pressed film coated with Chit-H
HC	PLA hot-pressed film coated with Chit-H/clove oil
HA	PLA hot-pressed film coated with Chit-H/argan oil
LC	PLA hot-pressed film coated with Chit-L/clove oil
LA	PLA hot-pressed film coated with Chit-L/argan oil
	**Coated Samples: PLA-NATIVIA Substrate**
HC-NATIVIA	Commercial PLA-NATIVIA^®^ NTSS 40 µm coated with Chit-H/clove oil
HA-NATIVIA	Commercial PLA-NATIVIA^®^ NTSS 40 µm coated with Chit-H/argan oil
H-NATIVIA	Commercial PLA-*N*ATIVIA^®^ NTSS 40 µm coated with Chit-H

**Table 2:** Low and high values (range) of the particle and fiber diameter and height distributions evaluated from AFM images.

Sample	Range of the Particle Diameter Distribution (nm)	Range of the Fiber Diameter Distribution (nm)	Range of the AFM Height Distribution (nm)
**HA**	20–140	20–140	150–500
**HC**	10–80	30–140	150–600
**LA**	10–60	-	10–55
**LC**	20–80	-	10–80

**Table 3:** Results of the antibacterial tests performed according to ISO 22196:2007 (E) (only the upper face was treated).

Sample	Log Reduction of the Number of Viable Bacterial Cells	
	*E. coli*	*S. Aureus*
**PLA Hot-Pressed**		
PLA (uncoated)	invalid	−0.8
HC	invalid	0.8
HA	invalid	0.7
**PLA-NATIVIA^®^ NTSS 40 µm**		
NATIVIA (uncoated)	1.1	−0.7
H-NATIVIA	1.8	0.8
HC-NATIVIA	2.2	1.2
HA-NATIVIA	0.8	0

**Table 4:** Results of the antibacterial tests performed according to ISO 16649-2/2007.

Sample	*Escherichia* *Coli*		*Listeria* *Monocytogenes*		*Salmonella* *Typhymurium*	
	24 h	48 h	24 h	48 h	24 h	48 h
	Inhibition (%)					
**PLA Hot-Pressed**						
**PLA -H**	26	42	29	32	22	29
**LA**	43	70	49	54	37	49
**LC**	68	80	37	63	100	100
**HA**	50	77	60	**100**	51	78
**HC**	80	91	77	**100**	100	100
**PLA NATIVIA^®^ NTSS 40 µm ^1^**						
**H-NATIVIA**	10	53	16	58	35	71
**HA- NATIVIA**	49	82	47	**100**	55	94
**HC- NATIVIA**	53	78	53	**100**	65	90

^1^ As the antibacterial tests for the PLA hot-pressed samples evidenced the highest antimicrobial efficiency for the samples containing Chit-H, the commercial NATIVIA^®^ foils were coated only with formulations containing Chit-H.
